# Matrix Metalloproteinases in Ureteropelvic Junction Obstruction: Their Role in Pathogenesis and Their Use as Clinical Markers

**DOI:** 10.3390/cells14070520

**Published:** 2025-03-31

**Authors:** Giusi Alberti, Eleonora Russo, Melania Lo Iacono, Maria Rita Di Pace, Francesco Grasso, Fabio Baldanza, Marco Pensabene, Giampiero La Rocca, Maria Sergio

**Affiliations:** 1 Department of Biomedicine, Neurosciences and Advanced Diagnostics (BiND), University of Palermo, 90127 Palermo, Italy; giusi.alberti@unipa.it (G.A.); melania.loiacono@unipa.it (M.L.I.); 2 Departmental Faculty of Medicine, Saint Camillus International University of Health Sciences, 00131 Rome, Italy; eleonora.russo@unicamillus.org; 3 Department of Health Promotion, Mother and Child Care, Internal Medicine and Medical Specialties “G D’Alessandro”, University of Palermo, 90127 Palermo, Italy; mariarita.dipace@unipa.it (M.R.D.P.); ciccio-grasso@hotmail.it (F.G.); fabio.baldanza@gmail.com (F.B.); marco.pensabene@policlinico.pa.it (M.P.)

**Keywords:** ureteropelvic junction obstruction, children, animal models, matrix metalloproteinases, metalloproteinase-specific inhibitors

## Abstract

The obstruction of the urinary tract is responsible for obstructive nephropathy (ON), also known as uropathy, which may then evolve in a renal parenchymal disease (hydronephrosis). Regarding the etiology of ON, it has been linked to the perturbation of processes occurring during the urinary tract development such as morphogenesis, maturation, and growth. Despite the research carried out in recent years, there is still a pressing need to elucidate the molecular processes underlying the disease. This may then result in the definition of novel biomarkers that can help in patient stratification and the monitoring of therapeutic choices. Matrix metalloproteinases (MMPs) are a family of zinc-dependent endopeptidases with key roles in extracellular matrix remodeling due to their wide cleavage specificity and ability to modulate the bioavailability of growth factors. Despite the known changes in the local tissue microenvironment at the site of the urinary tract obstruction, the role of MMPs in ureteropelvic junction obstruction (UPJO) and, therefore, in the pathogenesis of renal damage in ON is not well-documented. In this review, we underline the possible roles of MMPs both in the pathogenesis of UPJO and in the progression of related hydronephrosis. The potential use of MMPs as biomarkers detectable in bodily fluids (such as the patient’s urine) is also discussed.

## 1. Introduction

Obstructive nephropathy (ON), also known as uropathy, is a condition that can be induced by an obstruction of the urinary tract with the consequent development of renal parenchymal disease (hydronephrosis). The causes of this disease can be multiple: structural constriction of the ureteral lumen, impaired ureteral smooth muscle development affecting peristalsis, as well as secondary ureteral injury with renal failure [1,2]. Notably, ON is often a consequence of the impairment of various renal processes that occur during early development, including morphogenesis, maturation, and growth. Urinary tract development is modulated by a network of interactions between genetic and non-genetic actors, which are not yet fully understood [3]. Congenital obstructive nephropathy (CON) is one of the three chief etiologies causing pediatric chronic kidney disease (CKD). The most frequent pathophysiological cause of neonatal hydronephrosis is ureteropelvic junction obstruction (UPJO), which has an incidence of 1:1500 and a male-to-female ratio of 2:1 in the neonatal population [4]. Despite the numerous clinical and experimental studies conducted in recent decades on animal models [5,6,7,8,9], the necessary information on the timing of surgery and the prediction of disease progression continue to be lacking, thus remaining an important challenge for clinicians. For this reason, the assessment and management of fetuses, infants, and young children with CON remain an open question. Currently, biomarkers used to manage CON comprise ultrasound measurements, diuretic renography, and assessment of glomerular and tubular blood function (i.e., serum creatinine concentration). However, these parameters perform poorly in distinguishing obstructive cases from non-obstructive ones. For some authors, the identification of the number of functioning nephrons could be predictive of the functional outcome or could be used as a prognostic indicator [10,11,12,13]. Nevertheless, none of the previous studies are sufficiently strong or conclusive in clinically confirming the presence of ON in the prenatal phase and monitoring it later in the child’s life. Further elucidation of the molecular processes underlying the disease could help in the determination of early biomarkers that can help direct the therapeutic decisions necessary to prevent lesion progression and improve kidney remodeling after surgical correction of the obstruction.

Matrix metalloproteinases (MMPs) are a family of zinc-dependent endopeptidases capable of degrading and remodeling the proteins that form the extracellular matrix (ECM). Due to the broad range of potential substrates and the presence of several gene products belonging to the MMP family, they can contribute in a dual manner to the context-dependent fibrotic process within renal tissue [14]. MMPs are implicated in kidney formation processes (e.g., in the development of nephrons), as well as in adult kidney function. This further implies that altered secretion/distribution/activity of these enzymes could lead to the development and eventually the progression of a pathological condition. The possible roles of MMPs in UPJO and, therefore, in the pathogenesis of renal damage in obstructive nephropathy are not widely documented in the literature [15,16,17,18]. It has been observed that MMPs can stimulate renal fibrosis even during the evolution of post-obstructive renal damage by favoring an epithelial–mesenchymal transition (EMT) [19]. In addition, a recent study focused on the assessment of MMPs in the urine of 40 children with unilateral non-obstructive UPJO over a two-year timeframe [20].

In this review, we examined the literature on UPJO in order to highlight the identification of potential biomarkers that may be normally expressed during the development of the urinary tract and participate in the etiological phase of UPJO. The possibility of using these potential markers for the correct classification and stratification of patients and the possible clinical implications are also discussed.

## 2. Etiology and Features of UPJO

The current view of UPJO as the cause of neonatal hydronephrosis points to a multifactorial and polygenic etiology of obstructive damage. From a genetic point of view, few mutations have been tentatively linked to ON onset. For example, it has been reported that in a familial history case of UPJO, there were mutations in Wilms tumor genes [21]. Nevertheless, apart from these cases, it is very difficult to link the etiology of obstructive disease to single genetic factors. Once UPJO is established, during the early development of the renal tract, disease progression leads to tubulointerstitial inflammation and fibrosis, both of which affect the growth of the obstructed kidney, resulting in the compensatory expansion of the contralateral kidney. The outcome of severe ON is a continuous renal tubular atrophy alongside interstitial fibrosis with loss of nephrons [22]. The succession of the renal response to urinary tract obstruction and tubular expansion involves a chain of events resulting in impaired regulation of intrarenal components of the renin–angiotensin system (RAS), changes in inflammatory processes, etc. [23,24]. Furthermore, the clinical course of each patient is influenced by intrinsic factors related to specific genetic and non-genetic features. Differences may be related to the developmental phase wherein the obstruction originated, alongside the degree and timing of the occlusion and its position [22]. Accordingly, clinical indications focus on reducing obstructive renal damage and improving long-term outcomes by surgical correction [25].

### 2.1. Pathological Classification

Based on the origin of the obstructive pathology, UPJO can be (i) intramural, (ii) mural, or (iii) extramural. The most frequent obstruction is the “mural” one, caused by pathological alterations both in the rearrangement of smooth muscle cells and in the pyeloureteral innervation at the level of the ureteral wall. Both processes may then lead to a narrowing of the lumen. Other reports point to the role of extrinsic compression of the proximal ureter by the presence of accessory lower pole renal vessels, or by intrinsic anomalies. More rarely, the etiology of the occlusion may be the consequence of an atypical origin of the ureter from the pelvis [26]. Both extramural and intramural obstructions are less common in newborns [26]. UPJO is also classified as primary (congenital) and secondary (acquired). Congenital UPJO is considered a predominant cause of chronic kidney disease, leading to damage of the kidneys, ureter, and/or bladder linked to urinary tract obstruction, and originates from unilateral or bilateral malformations [27]. It is typically assessed based on the presence of prenatal hydronephrosis, with disorders that vary depending on the severity of the ureteropelvic junction [27]. Most UPJO cases are caused by partial obstruction of the ureteropelvic junction, since total obstructions can lead to rapid renal failure [27]. The causes of acquired UPJO include external and/or internal factors. Acquired UPJOs are infrequent and often linked to vascular compression or retroperitoneal fibrosis, while intrinsic UPJOs are linked to chronic inflammation, failed repair of a primary UPJO, and other factors [28]. In addition, UPJO can be classified as acute or chronic, unilateral or bilateral, and partial or complete [29,30].

### 2.2. Functional Obstruction Causes

Defects in the evolution of the smooth muscle lining the pelvis and ureter may be implicated in the functional obstruction giving rise to the pathogenesis of UPJO [31,32]. Around the 12th week of human gestation, the mesenchymal layer, which overlies the epithelial layer in the ureteral tree, guided by a sequence of autocrine and paracrine signals, differentiates into smooth muscle, which is responsible for the generation of the peristaltic waves that expel urine [31,33].

Histopathological studies of the ureteropelvic junction of patients undergoing pyeloplasty (i.e., surgical reconstruction or revision of the renal pelvis to drain and decompress the kidney) have highlighted the formation of smooth muscle hypertrophy along with perifascicular fibrosis, as well as inflammatory cell infiltration [34,35]. Although this condition can vary in severity from patient to patient, it is known that the premature evolution of the obstruction during nephrogenesis in utero leads to a worse prognosis [36].

Regarding the modifications in renal parenchyma, histological studies allowed identifying the main pathological changes in UPJO patients: glomerulosclerosis, widening of Bowman’s capsule, interstitial fibrosis, and tubular atrophy, the evaluation whereof has been proposed as a predictor of renal function recovery following successful corrective surgery [37,38]. It follows that, although surgery in UPJO obstruction is effective in protecting against renal injury detectable in the short term, it remains necessary to understand which changes in the renal parenchyma cannot be reverted using the surgical approach, either for timing reasons or for the need of an improved clinical procedure. This has led to the development of animal models to gain deeper insight into the disease etiology and its pathophysiological features both at the level of the primary obstruction and in the renal parenchyma.

## 3. Animal Models to Study Ureteral Obstruction

In the last few decades, several animal models of human UPJ obstruction have contributed to supporting the theory that “the earlier the obstruction occurs during nephrogenesis, the more acute the associated histopathological changes” [7,39,40,41]. In this regard, the studies reported alterations in renal expansion, nephron number, and glomerular and tubulointerstitial histology, both in the affected and in the contralateral kidney (i.e., non-obstructed) [7,40,41,42,43]. These features lead to a practical difficulty in identifying the condition and, therefore, surgery remains the primary treatment choice in cases of urinary tract obstruction.

In the 1970s, the first unilateral ureteral obstruction (UUO) model was defined in rabbits, thus opening up the possibility for researchers to use animal models to elucidate the pathogenesis of obstructive nephropathy [44] (Table 1). Studies of the consequences of long-term urinary tract obstruction, particularly unilateral partial ureteral obstruction (PUUO), have also begun more recently [45,46,47]. A newborn guinea pig model of PUUO was particularly useful in appreciating the link between the damage induced by the obstruction and the processes of renal development. In guinea pigs, the extent of nephrogenesis at birth is almost identical to that of the second-trimester human fetus. It has been reported that the obstruction interferes with renal morphogenesis, growth, and maturation, although embryological breakdown probably occurs much earlier [45]. In this study, the obstruction level reflects the most commonly found clinical condition in cases of UPJO, i.e., partial rather than complete obstruction [45] (Table 1).

However, complete unilateral ureteral obstruction (CUUO) has been classically considered the model of choice for ON to explore in depth how obstruction of the UPJ affects renal histology and consequently renal function, thus allowing the identification of potential biomarkers involved in the pathology [47]. In comparison with the PUUO model, the CUUO model is less difficult to set up surgically, resulting in reduced variability in the experimental results. This result has its limits in that this model is not able to finely reproduce the changes induced by inflammation or fibrosis in the renal parenchyma. Nonetheless, CUUO models significantly alter renal blood flow, as well as glomerular filtration and tubular activity. Morphological variations in this model include subtle anatomical changes in blood vessels, glomeruli, and tubules [48]. Although in many instances clinical ON may be more consistent with the features of the CUUO model, it should be noted that the pattern of complete obstruction mimics a condition that is not present, except occasionally, in human disease. Conversely, the PUUO models show better similarity to the condition that develops in humans, and, therefore, the timing of disease onset, as well as the appearance of urinary biomarkers, could be more comparable to those actually observable in children. Nevertheless, the PUUO model is technically challenging, with recurrent adhesions leading to absolute obstruction [46,49,50,51,52]. These studies, even without recapitulating entirely the features of multifaceted human disease, guarantee the possibility of overseeing the onset, the development, and the severity of the obstruction. Instead, bilateral ureteral obstruction (BUO) is a model frequently used to examine the pathophysiological role of transport proteins [46] (Table 1).

## 4. Pathological Changes in Obstructive Nephropathy at the Tissue and Molecular Levels

**Figure 1:**
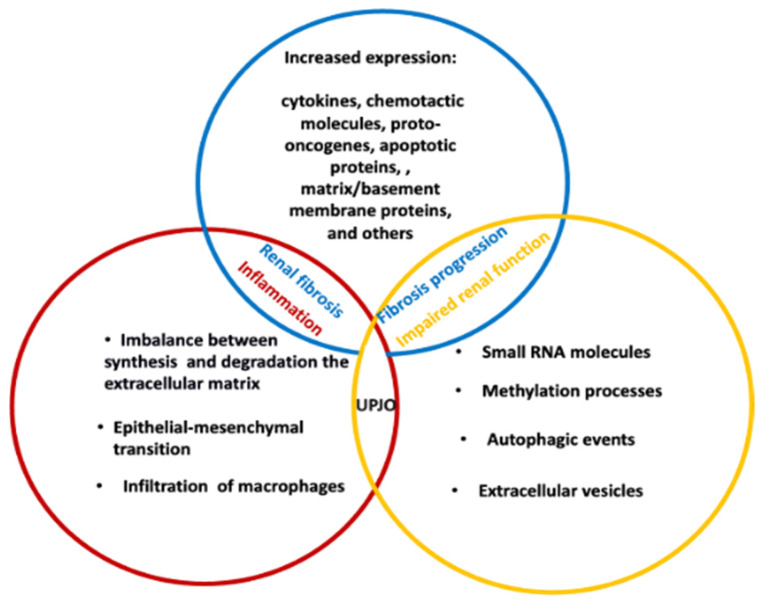
The pathogenesis of renal damage and the progression of ON depend on a series of events at the cellular level that include renal tubular dysfunction, phenotypic cell change, and interstitial inflammation, followed by interstitial fibrosis. This process involves many enzymes, cytokines, signaling molecules, miRNAs, and EVs.

ON in children depends on the cellular interplay in which a variety of locally and systemically active molecules act as paracrine intercellular mediators [53,54]. ON can induce important modifications in the tubulointerstitial compartment of the kidney, the extent of impairment being contingent on the duration and severity of the obstruction. This is regardless of the lesion being either unilateral or bilateral, acute or chronic [55,56]. A common and key consequence of ON is renal interstitial fibrosis, probably reflecting a disparity between the processes of synthesis and deposition of the extracellular matrix (ECM) and the degradation of the ECM itself [57]. As early as 1973, Nagle et al. reported an increase in collagen fibers and fibroblasts after 7 days of chronic unilateral obstruction in rabbits, with significantly increased collagen deposits [58]. Furthermore, the same authors identified the presence of mononuclear infiltrates, as well as an increase in interstitial cells in the renal parenchyma in this animal model [58]. Sharma et al. also observed that interstitial fibrosis was related to a thickening of the basement membrane of the renal tubules following unilateral obstruction in the rabbit. The authors suggested that the process was driven by deposits of ECM components such as collagens (types I, III, and IV) as well as fibronectin after 3, 7, and 16 days [59]. In addition, the increase in interstitial fibrosis is also linked to the contribution of renal tubular epithelial cells, which undergo epithelial–mesenchymal transition (EMT), generating further fibroblastoid populations [60]. Other studies have highlighted an increased recruitment of interstitial macrophages, together with augmented fibroblasts, which are processes probably triggered by the secretion of chemoattractant proteins, such as monocyte chemoattractant protein-1 (MCP-1) and tumor necrosis factor-α (TNF-α). In this process, the activity of protease-activated receptor-1 (PAR-1) was involved in driving the EMT of tubular epithelial cells in mice with UUO [60,61] (Figure 1). These processes may enhance tissue damage by promoting the apoptosis of epithelial cells, endothelial cells, and podocytes, leading to renal hypoxia and ischemia. This outcome leads to the lack of peritubular capillaries and glomeruli, as well as the loss of proximal tubules. This contributes to the generation of “atubular glomeruli” with consequent tubular atrophy [62,63]. Phenotypic transitions occur in epithelial cells (EMT), endothelial cells (EndMT), pericytes, or in the differentiation of stem cells. These processes generate new fibroblasts, which also present the features of myofibroblasts, such as the expression of α-smooth muscle actin (α-SMA). These cells participate in the altered deposition/remodeling of the ECM and, therefore, in the progression of interstitial fibrosis. A crucial outcome of these events is the gradual loss of the normal constituents of the nephron [25,62,63]. All these mechanisms, also associated with the structural imbalance of smooth muscle, form the basis of UPJO, although further studies are needed to confirm these biological/pathological aspects.

Literature reports focus on a plethora of molecules whose expression is altered in newborns, infants, and children with prenatally-detected hydronephrosis, including a number of cytokines, chemotactic molecules, proto-oncogenes, apoptotic proteins, matrix/basement membrane proteins, and more [64,65] (Figure 1). Furthermore, it has been demonstrated that the impairment of epithelial cells downregulates epidermal growth factor (EGF) and triggers the local renin–angiotensin system that prompts the downstream expression of various factors, such as transforming growth factor-β1 (TGF-β1), a key transforming factor involved in the renal fibrosis process. This alteration also involves tumor necrosis factor-α (TNF-α), vascular cell adhesion molecule 1 (VCAM-1), and nuclear factor kB (NF-kB), besides contributing to the development of reactive oxygen species (ROS) [66,67,68]. In turn, TGF-β1 action is reflected in increased interstitial fibrosis by inhibiting extracellular MMPs, as well as upregulating the expression of tissue inhibitors of metalloproteinases (TIMPs). In fact, one study described a crucial impairment in the expression of TIMP-3 in the cortex of the obstructed kidney, although further experiments are necessary to substantiate the biological implications of these proteins [69]. Furthermore, the evolution of tubular injury is also closely linked to the de novo expression of several keratin isoforms (e.g., keratin 5) in damaged proximal tubule epithelial cells. These may represent the very first signs of the developing disease when no signs of fibrosis are yet detectable [70]. The function of Akt1 in renal fibrosis has also been considered: Akt1 is able to mediate the transforming growth factor β1 (TGF-β1)/transcriptional activator 3 (STAT3) pathway to stimulate renal fibrosis in mice [71].

Small RNA molecules, methylation processes, as well as autophagic events of intercellular communication mediated by extracellular vesicles (EVs) also intervene in the development of UPJO [72,73,74,75] (Figure 1). It has been reported that the dysregulation of a few miRNAs, such as miR-21 and miR-29, is connected with the evolution of renal fibrosis in UPJO pathogenesis [76,77]. For instance, increased miR-21 was identified in animal and human samples, in both urine and kidney tissue, during renal fibrosis [78,79] (Figure 1). EVs are vesicles with a diameter ranging between 100 and 200 nm, released by cells into the extracellular environment. Their cargo is represented by proteins, nucleic acids, and lipids, so that EVs may intervene in cellular communication and the transfer of genetic information, reflecting and regulating physiological and pathological processes [80,81,82,83,84,85]. Urine may contain significant quantities of EVs since both intrinsic renal cells and renal tubular epithelial cells and podocytes are capable of releasing EVs [86,87]. It has been observed that renal tubular epithelial cells release miR-21-enriched EVs capable of activating fibroblasts in obstructed kidneys via the miR-21/PTEN/Akt pathway, thus promoting the evolution of renal fibrosis [66]. Furthermore, it has been shown that decreasing the expression of exosomal miR-26a in kidneys of UUO mice reduces the protein levels of two pro-fibrotic proteins, such as connective tissue growth factor (CTGF) and TGF-β1 in the kidneys of UUO mice, thus limiting renal fibrosis through CTGF inhibition [88].

Taken together, all the above studies suggest that the dysregulation of molecules/proteins involved in specific signaling pathways, as well as the release of EVs enriched with bioactive factors (and potential biomarkers), contribute through various processes to the onset of renal fibrosis, a factor linked to the development of UPJO.

## 5. MMPs as Potential Biomarkers in Infants with UPJO

### 5.1. MMP Structure, Classification, and Function

MMPs are a family of zinc-dependent endopeptidases capable of degrading all the molecular components of the ECM and of cleaving a wide range of substrates, including cell surface receptors and adhesion molecules, binding proteins, growth factors, and cytokines. Their widespread action renders MMPs crucial players in regulating ECM remodeling and controlling many cellular processes, including cell proliferation, migration, differentiation, angiogenesis, and apoptosis [89]. The canonical structure of the MMPs is characterized by a proenzyme and catalytic domain, a hinge region, and a hemopexin-like domain [89,90], and they are conventionally classified according to their structure and/or ECM substrate specificity into four principal groups: interstitial collagenases, gelatinases, stromelysins, and membrane-type (MT)-MMPs [91,92]. MMPs are expressed in both developing and adult kidneys, and they are involved in the regulation of nephron formation as well as in the pathogenesis of kidney damage [91,93,94]. It has been reported that MMPs are secreted by resident glomerular and tubular epithelial cells [95,96]. Considering their proteolytic potential, MMPs are commonly described as antifibrotic players in the development and advancement of CKD, in which tissue fibrosis is one of the expected consequences [83,86,87]. The activity of MMPs is controlled by a family of naturally occurring endogenous inhibitors known as tissue inhibitors of metalloproteinases (TIMPs). The four known TIMPs have specificity for distinct MMPs, yet collectively they can inhibit them all [95]. Furthermore, dysregulation in the expression and levels of MMPs or their inhibitors has been associated with structural variations that appear in the evolution and progression of kidney disease [97,98,99]. In nephropathies, MMPs seem to play a dual role as antifibrotic molecules and as proinflammatory mediators. Nonetheless, the correct biological role in a given renal disease might be conditional on the expression levels as well as MMP activity, together with the severity or the occurrence of the respective disorder.

MMP-2 and MMP-9 are named gelatinases because they are capable of degrading denatured collagen following the proteolytic cuts promoted by proper collagenases (e.g., MMP-1) on collagen fibrils. Moreover, gelatinases feature type IV collagen and laminin as specific targets of their proteolytic activity. These enzymes are expressed at various levels in the renal parenchyma, including the glomeruli, the proximal and distal tubules, and the collecting ducts [100,101]. Furthermore, they play key roles in the recruitment and chemotaxis processes of inflammatory cells [102]. In addition, MMP-2 represents an effective activator of MMP-1 and MMP-9 through the cleavage of their prodomains [103]. MMP-1 is an interstitial collagenase capable of degrading native collagen and, therefore, its function as an antifibrotic enzyme is hypothesized, even though its role in renal diseases is not fully known [104,105]. MMP-1, together with MMP-2, MMP-9, TIMP-1, and TIMP-2, participates in the remodeling processes of the ECM within the kidney interstitium [106,107]. TIMPs are endogenous inhibitors of metalloproteinases and, therefore, represent key regulators of extracellular matrix turnover, as well as of tissue remodeling and cellular behavior [108]. Specifically, TIMP-1 has a narrower inhibitory specificity compared to the other three TIMPs. TIMP-2 and -3 are weaker inhibitors than TIMP-1 for MMP-3 and MMP-7, but not for the other MMPs [109].

### 5.2. Roles of MMPs in UPJO Development and Progression

Several studies have shed light on the role of specific MMPs in the regulation of physiological homeostasis and pathological disorders, currently poorly explored [93,97,98,99]. MMPs are expressed in both developing and adult kidneys, and they are involved in regulating nephron formation, as well as in the pathogenesis of many kidney disorders [110,111]. Given their proteolytic potential, MMPs are considered important antifibrotic actors during the evolution and progression of CKD, particularly considering the features typically associated with UPJO [95,96,110]. This consideration, however, needs to be carefully weighed, since new paradigms are emerging on the critical roles of MMPs in the onset of a variety of fibrotic kidney disorders [112,113].

Dysregulation of the expression levels and functions of MMPs and TIMPs has been documented in the renal tissue of rats affected by hydronephrosis [69,114] (Table 2).

In renal fibrosis, the contribution of MMP-9 has been evaluated very thoroughly. In vitro reports have documented that MMP-9 is capable of promoting the EMT process of renal tubular cells through the disruption of the cell–cell adhesion complex, mediated by E-cadherin/b-catenin. The molecule is also detectable in the serum of mice that have undergone the development of unilateral ureteral obstruction (UUO) [115,116] (Table 2). Nonetheless, these reports have several limitations due to the simplification of the biological complexity in MMP-9 knockout mice. However, another study confirmed the important activity of MMP-9 in the development of renal fibrosis during the evolution of post-obstruction renal damage as a result of the stimulation of the EMT [117] (Table 2). In particular, MMP-9 may participate in the development of this condition via the Notch signaling pathway in primary renal peritubular endothelial cells and glomerular endothelial cells of mice and humans, respectively [118,119] (Table 2). Currently, it is known that mesenchymal stem cells may play a protective role against obstruction-induced renal fibrosis by deregulating MMP-9 production [124]. Notably, MMP-9 contributes to kidney fibrosis by stimulating the recruitment of macrophages and the cleavage of osteopontin [125]. In addition, there is a positive association between the serum levels of MMPs and the process of kidney fibrosis. Indeed, a study conducted on children with unilateral non-obstructive UPJO highlighted the presence of MMP-2 in serum as a potential noninvasive biomarker [120] (Table 2). On the contrary, some authors have demonstrated that an increase in the expression of MMP-9 constitutes an indicator of the success of surgery in children with UPJO as it leads to an increase in degradation processes in the ECM [126]. Recently, Bieniaś et al. (2020) showed an increase in the concentration of MMP-9 in serum and urine, as well as decreased serum MMP-9/TIMP-1 and MMP-9/TIMP-2 ratios in patients with UPJO, which may be important for the development of renal fibrosis [127]. Similarly, to MMP-9, the expression of MMP-2 was also studied in 21 children who had undergone dismembered pyeloplasty using immunostaining analysis [121]. In this work, the authors highlighted the replacement of smooth muscle tissue with connective tissue, together with an increase in the expression of MMP-2, indicating an incessant turnover of the extracellular matrix compared to the healthy controls [121] (Table 2). Furthermore, it was observed that MMP-2 knockout and heterozygous mice were protected from renal fibrosis despite the presence of coexisting UPJO [115]. Additionally, another study suggested that urinary secretion of MMP-2 and MMP-9 is closely related to the regulation of transforming growth factor beta (TGF-β), thus confirming their profibrotic action [16]. Recently, significantly higher concentrations of MMP-2, together with TIMPs, have been further confirmed in the urine of children with unilateral UPJO [121,122] (Table 2). Of note, elevated levels of TIMP-2 in the urine were correlated with the severity of the obstruction, representing a useful biomarker for correctly managing children between surgical treatment and follow-up. However, after pyeloplasty, the levels of TIMPs in the urine showed a progressive decrease, thus further suggesting their usefulness as urinary markers [122,123]. These findings highlight a correlation between UPJO severity, accumulation of the ECM in the renal parenchyma, and the development of tubulointerstitial fibrosis. Recently, a study revealed a promising role of MMP-7 and TIMP-2 for the selective stratification and management of patients affected by UPJO [124] (Table 2). The results of this work demonstrated that high urinary levels of MMP-7 and low levels of TIMP-2 constitute a useful combination for identifying impaired renal function in a heterogeneous population of patients with UPJO regardless of etiology or clinical severity [124]. Furthermore, high concentrations of TIMP-2 were found in the urine of patients with UPJO compared to the control group, while no difference was observed between the groups of patients with or without obstruction [128]. Another study described the possibility of using TIMP-2 as a biomarker of obstruction [129].

### 5.3. Dysregulation of MMPs and TIMPs in UPJO

**Figure 2:**
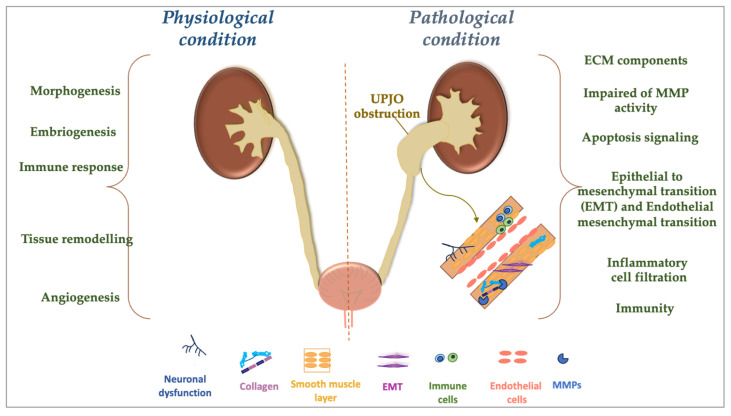
Cartoon representing the consequences that UPJO can trigger in children or adults during the prenatal and/or postnatal period. On the right, the figure identifies the pathological condition of UPJO with the dilation of the pelvis of the kidney (hydronephrosis), accompanied by the onset of a plethora of processes, including an increase in the ECM components, impaired MMP activity, and the induction of apoptotic processes, with a consequent increase in infiltration by immune cells, as well as the development of the EMT together with the EndoMT. On the left, the figure represents the physiological condition that can be observed in the urinary system of a healthy subject.

Although several reports are already available in the literature, further research is needed to identify children with UPJO at an early stage to assist with surgical decision-making in the future. Currently, a large number of urinary and serum biomarkers have been investigated, belonging to different protein families such as TGF-β, TNF-α, epidermal growth factor (EGF), angiotensinogen, ficolin-2, as well as immunoglobulin superfamily containing leucine-rich repeat and other molecules [130,131,132]. As suggested above, alone or in combination with TIMPs, MMPs may also help to identify impaired renal function in children with UPJO. The main pathological features of UPJO are linked to altered collagen deposition by muscle cells, an abnormality that exacerbates an inflammatory response and other conditions at the renal level [35,57,58] (Figure 2).

In this regard, an increase in extracellular matrix proteins within the smooth muscle compartment and the stromal matrix has been reported in human UPJO samples. In particular, the deposition of type IV collagen and laminin might be associated with an increased expression of MMP-2 and MMP-9 [112,133] (Figure 2). A further increase in the turnover of type III collagen deposition in the ECM in UPJO is mediated by MMP-1 and MMP-2 [19,119]. Impaired MMP-2 activity and TIMP-2 deficiency may lead to the development of fibrosis, renal tubular atrophy, and ECM deposition, which result in the progression of the lesion [134]. The latter causes the ECM degradation and the evolution of chronic inflammation that worsens the damage [134]. The rapid development of renal fibrosis is further attributable to the ability of MMP-2 to degrade the basement membrane, as well as its ability to alter glomerular filtration and favor the phenotypic transformation of tubular epithelial cells [135]. MMP-7 may also participate in renal fibrosis by promoting the EMT and increasing the ECM deposition [136]. In addition, increased MMP-9 expression, in parallel with the decrease in TIMP-1 levels, results in an alteration of the ECM homeostasis, leading to renal fibrosis [135]. Nonetheless, increased TIMP-1 expression is induced by TGF-β1 [137]. The fibrotic process is a key component of the EMT activation that causes the migration and invasion of newly formed mesenchymal cells into the interstitial space, resulting in the progression of fibrosis through the deposition of the extracellular matrix. Both MMP-2 and MMP-9 play a crucial role in this process [134,135,137] (Table 2 and Figure 2). Literature reports have demonstrated that the induction of the EMT in tubular cells is associated with an increased expression of MMP-2 and MMP-9 [138,139]. Increased MMP-9 expression promotes disruption of the tubular basement membrane integrity and facilitates the tubular EMT following stimulation by tissue-type plasminogen activator (TPA) [139,140]. In addition, various cell types, such as mesangial, epithelial, endothelial, as well as tubular cells of the collecting duct and fibroblasts, express high levels of MMP-2 and MMP-9, which could directly trigger the entire process of the renal tubular cell EMT [118,134]. The perpetuation of the MMP-2 and MMP-9 activity was also related to the activation of resident fibroblasts, endothelial–mesenchymal transition (EndoMT), and pericyte–myofibroblast trans-differentiation [141].

Studies have reported apoptosis or atrophy events of smooth muscle cells in UPJO patients, resulting in the observation of thin muscle fibers immersed in a rich collagen network, intertwined with missing neural components [142,143,144] (Figure 2). MMPs and TIMPs can intervene in intracellular signaling in apoptosis processes [145]. In fact, obstruction of the urinary flow with a consequent increase in intraparenchymal pressure activates a sequence of inflammatory and oxidative events at the tubulointerstitial level. This increases the hydrostatic pressure and causes a progressive swelling of tubular cells, which can trigger downstream apoptotic processes [146] (Figure 2). Importantly, it has been observed that tubular injury affects primarily the distal and collecting tubules, although the proximal tubular compartment is also subject to changes [147]. In this context, MMP-2 may promote the activation of zymogens MMP-1 and MMP-9 to create a direct association between fibrosis and inflammation at the tubulointerstitial level. It has been reported that MMP-2 is secreted in different regions of the nephron (e.g., by glomerular mesangial cells, renal tubular epithelial cells, and interstitial cells), playing important functions underlying the progression of the disease [135]. Additionally, MMP-2 and MMP-9, expressed in different renal parenchymal areas, are essential for the recruitment and chemotaxis of inflammatory cells [135,148]. In addition, an imbalance between TIMPs/MMPs could not only lead to fibrosis, but also exacerbate an inflammatory condition [148]. Fibrotic and inflammatory changes are usually unrecoverable and, as in other cases of renal injury, the severity of fibrosis normally correlates with the size of renal damage.

## 6. MMP and TIMP Roles in Other Renal Conditions

It is well-known that several MMPs and TIMPs are expressed in the kidney, namely in the nephron compartments, in the connective tissue, and beyond, where they may be involved in physiological and pathological pathways affecting this organ, such as UPJO [103,108,149]. It is reported that the activity of variuos MMPs and TIMPs is altered in a wide variety of kidney disorders, including acute kidney injury (AKI), renal fibrosis (KF) (which is the most prevalent histological hallmark of CKD), diabetic kidney disease (DKD), nondiabetic glomerular disease, and polycystic kidney disease (PKD) [150]. Among these, KF and DKD represent two pathological conditions in which MMPs and TIMPs are involved not only in the ECM remodeling, but also in the release of several growth factors; thus, their dysregulation interferes with the normal ECM turnover. They are also involved in the pathological mesangial expansion, as well as in glomerulosclerosis and the stimulation of the EMT of renal epithelial cells, causing progressive development of the renal disease [150,151]. For example, La Russa et al. described how MMP-9 dysregulation underlies profibrotic changes in tubular epithelial cells following macrophage recruitment, triggered by proteolytic activation of osteopontin in renal fibrosis [94]. Moreover, the dysregulation of MMP-2 expression either promotes or reduces the deposition of the ECM in the early or advanced phases of renal fibrosis, respectively [136]. Additionally, both expression and activity of MMP-2 and MMP-9 are also altered in DKD [98]. Specifically, experimental studies have shown that these endopeptidases may play central roles in several inflammatory pathways made even more complex by subject-specific genetic heterogeneity [98]. It has also been observed that the concentrations/activity of both proteins are increased in the urine of type 1 and 2 diabetic patients [98,152]. Non-diabetic glomerular disease is poorly understood, but increased glomerular MMP-9 has been found in lupus nephritis, Henoch–Schoenlein purpura, and postinfectious glomerulonephritis [153]. PKD is the most common inherited kidney disease related to MMP and TIMP dysregulation. For example, serum from PKD patients has been found to be enriched in MMP-1, MMP-9, TIMP-1, and type IV collagen compared to the healthy controls [154]. Currently, organ transplantation remains a key choice for some kidney diseases, although it has several limitations related to chronic rejection and excessive costs, as well as the critical and controversial role of MMPs. Several studies have collectively identified increases in MMP-2, MMP-7, MMP-8, and MMP-9, as well as in TIMP-1 and -2 in cases of allograft rejection [155,156,157]. Recently, attention has been shifting either towards the use of mesenchymal stem cells or the use of immunosuppressive drugs (such as rapamycin), which are able to inhibit the expression of MMPs (such as MMP-9), thus increasing the expression of TIMPs (such as TIMP-1) [158,159]. As reported above, in these disorders, the role of MMPs and TIMPs is controversial as well.

## 7. Current Treatment of UPJO and Future Therapeutic Strategies

The surgical treatment of pyeloureteral junction stenosis has been well-standardized for several years. To date, the approach uses the classic open method in cases of PUJ obstruction in patients under 1 year of age and minimally invasive laparoscopic or robotic techniques in patients aged 12 to 18 months and older [160]. The technique consists in restoring normal patency to the stenotic segment following the principle of ureteropyelostomy and pyeloplasty according to Anderson–Hynes. The surgical outcome, in the hands of experienced surgeons, shows a very high success rate (95–98%), with a relatively low complication rate. Therefore, the issue that remains unresolved today is identifying patients at risk of requiring surgery in order to avoid delaying the intervention and worsening their renal condition or overestimating patients who should only be monitored conservatively. The available diagnostic tools are sometimes insufficient to estimate the actual renal damage, nor can they resolve the doubt between a functional or organic stenosis. In this context, metalloproteases, along with other factors involved in the renal cell turnover mechanism, could represent an important prognostic predictive factor both in the diagnostic timeframe and post-treatment. The study of such factors in urine would represent a minimally invasive and repeatable method to explore their behavior in selected populations. Nonetheless, further studies are needed to fully elucidate the role of MMPs in UPJO and develop targeted therapeutic strategies.

## 8. Conclusions

The theory that MMPs play a key role in the evolution of renal diseases is mainly based on the ability of MMPs to degrade the ECM components, as demonstrated by their altered expression in a large number of renal diseases [94,118,135]. Although there are few functional studies, it remains to be elucidated whether the dissimilar regulation of MMPs and TIMPs is a cause or simply an effect of the respective renal diseases. It follows that the correct biological function, in the context of a renal disease, may depend on the exact level of MMP and TIMP activity, the intensity or the chronicity of the disease itself. Assessing such factors in urine would represent a minimally invasive and repeatable method to explore their behavior in selected populations.

## Figures and Tables

**Table 1:** Animal models of UPJ obstruction.

Animal Models of UPJ Obstruction	Pathophysiology of Obstruction	References
Unilateral complete ureteral obstruction rabbit model	– Papillary necrosis within 7 days of obstruction with continued enlargement of the cortical interstitial area with a significant increase in fibroblasts – Sublethal cellular damage with loss of cellular specialization of the nephrons	[44]
Unilateral partial ureteral obstruction newborn guinea pig model	– Impaired somatic growth – Mean arterial pressure unchanged – Increased renal vascular resistance related to a decrease in perfused glomeruli with respect to the hypertrophic contralateral kidney – Decreased glomerular filtration rate	[45]
Bilateral ureteral obstruction young rat model	– Diminution of the single nephron glomerular filtration rate – Decrease in the reabsorption of Na and H_2_O before the bend of the loop of Henle – Alteration in fluid osmolality	[46]

**Table 2:** In vivo studies on the involvement of MMPs and TIMPs in UPJ obstruction.

MMPs and TIMPs	In Vivo Studies	UPJ Obstruction Condition	Pathophysiological Aspects	References
TIMP-1, TIMP-2, TIMP-3	Rat model	UUO	(i) In obstructed kidneys, increased TIMP-1 mRNA leads to the upregulation of TGF beta 1 post-UUO (ii) Reduction in the TIMP-3 mRNA expression levels after 24, 48, and 96 h (iii) The expression level of the TIMP-2 gene remains unchanged	[69]
MMP-2, TIMP, TIMP-2	Rabbit model	UUO	(i) Increased expression of mRNA of MMP-2 (ii) Significant growth of TIMPs in the UUO samples at all times (iii) TIMP-2 mRNA expression is biphasic, with peaks at both day 3 and day 16 of UUO	[114]
MMP-2 and MMP-9	Mouse model	UUO	(i) Elevated MMP-2 expression showed more histological damage (ii) Inactivation of MMP-2 protects mice against hydronephrosis and kidney fibrosis after UUO (iii) Inhibition of MMP-9 decreases the EMT and the fibrotic process during the early stages of onset or in the chronic phase, but not during the evolution of fibrosis	[19,115]
MMP-2 and MMP-9	Fetal ovine model	CUO	(i) Urinary MMP-9 constitutes a highly significant predictor of renal fibrosis and of higher degrees of fibrosis (ii) Urinary MMP-2 is found to be notably associated with a high degree of fibrosis	[116]
MMP-9	Mouse model	UUO	(i) MMP-9 also promotes the EMT of peritubular endothelial cells and contributes to kidney fibrosis (ii) MMP-9 induces the EMT via Notch activation in kidney glomerular endothelial cells	[117,118]
MMP-2	Human model (median age of 1.5 months)	UPJ	(i) The levels of MMP-2 expression represent matrix turnover in obstructed UPJ segments	[119]
MMP-2	Human model (mean age of 103.2 months)	UPJ	(i) Elevated levels of MMP-2 as compared to the healthy controls (ii) MMP-2 induces the replacement of smooth muscle cells with connective tissue	[120]
MMP-1,-2,-9 and TIMP-1,-2	Human model (children, 1 month–18 years)	UPJO	(i) Elevated levels of urinary MMP-2, TIMP-1, and TIMP-2 (ii) Elevated urinary TIMP-2 levels correlate with the severity of obstruction (iii) Urinary TIMP-1 and TIMP-2 decrease after pyeloplasty	[121,122]
MMP-7 and TIMP-2	Human model (median age of 3.3 years)	UPJO	(i) Increased levels of urinary MMP-7 and TIMP-2 as a prognostic and diagnostic tool for UPJO children	[123]
